# Myricetin bioactive effects: moving from preclinical evidence to potential clinical applications

**DOI:** 10.1186/s12906-020-03033-z

**Published:** 2020-08-01

**Authors:** Yasaman Taheri, Hafiz Ansar Rasul Suleria, Natália Martins, Oksana Sytar, Ahmet Beyatli, Balakyz Yeskaliyeva, Gulnaz Seitimova, Bahare Salehi, Prabhakar Semwal, Sakshi Painuli, Anuj Kumar, Elena Azzini, Miquel Martorell, William N. Setzer, Alfred Maroyi, Javad Sharifi-Rad

**Affiliations:** 1 Phytochemistry Research Center, Shahid Beheshti University of Medical Sciences, Tehran, Iran; 2 Department of Pharmacology and Toxicology, School of Pharmacy, Shahid Beheshti University of Medical Sciences, Tehran, Iran; 3 Department of Agriculture and Food Systems, The University of Melbourne, Melbourne, Australia; 4 Faculty of Medicine, University of Porto, Alameda Prof. Hernâni Monteiro, 4200-319 Porto, Portugal; 5 Institute for Research and Innovation in Health (i3S), University of Porto, 4200-135 Porto, Portugal; 6 Department of Plant Biology Department, Taras Shevchenko National University of Kyiv, Institute of Biology, Volodymyrska str., 64, Kyiv, 01033 Ukraine; 7 Department of Plant Physiology, Slovak University of Agriculture, Nitra, A. Hlinku 2, 94976 Nitra, Slovak Republic; 8 Department of Medicinal and Aromatic Plants, University of Health Sciences, 34668 Istanbul, Turkey; 9 Faculty of Chemistry and Chemical Technology, Al-Farabi Kazakh National University, Almaty, Kazakhstan; 10 Noncommunicable Diseases Research Center, Bam University of Medical Sciences, Bam, Iran; 11 Student Research Committee, School of Medicine, Bam University of Medical Sciences, Bam, Iran; 12 Department of Biotechnology, Graphic Era University, Dehradun, Uttarakhand 248001 India; 13 Uttarakhand State Council for Science and Technology, Vigyan Dham, Dehradun, Uttarakhand 248007 India; 14 Himalayan Environmental Studies and Conservation Organization, Prem Nagar, Dehradun, Uttarakhand 248001 India; 15 Uttarakhand Council for Biotechnology, Silk Park, Prem Nagar, Dehradun, Uttarakhand 248007 India; 16 CREA-Research Centre for Food and Nutrition, Via Ardeatina 546, 00178 Rome, Italy; 17 Department of Nutrition and Dietetics, Faculty of Pharmacy, and Centre for Healthy Living, University of Concepción, 4070386 Concepción, Chile; 18 Unidad de Desarrollo Tecnológico, UDT, Universidad de Concepción, 4070386 Concepción, Chile; 19 Department of Chemistry, University of Alabama in Huntsville, Huntsville, AL 35899 USA; 20 Aromatic Plant Research Center, 230 N 1200 E, Suite 100, Lehi, UT 84043 USA; 21 Department of Botany, University of Fort Hare, Private Bag X1314, Alice, 5700 South Africa; 22 Zabol Medicinal Plants Research Center, Zabol University of Medical Sciences, Zabol, Iran

**Keywords:** Myricetin, Antimicrobial, Antioxidant, Neuroprotection, Diabetes, Cancer, Immunomodulatory, Cardiovascular disease

## Abstract

Several flavonoids have been recognized as nutraceuticals, and myricetin is a good example. Myricetin is commonly found in plants and their antimicrobial and antioxidant activities is well demonstrated. One of its beneficial biological effects is the neuroprotective activity, showing preclinical activities on Alzheimer, Parkinson, and Huntington diseases, and even in amyotrophic lateral sclerosis. Also, myricetin has revealed other biological activities, among them as antidiabetic, anticancer, immunomodulatory, cardiovascular, analgesic and antihypertensive. However, few clinical trials have been performed using myricetin as nutraceutical. Thus, this review provides new insights on myricetin preclinical pharmacological activities, and role in selected clinical trials.

## Introduction

**Fig. 1:**
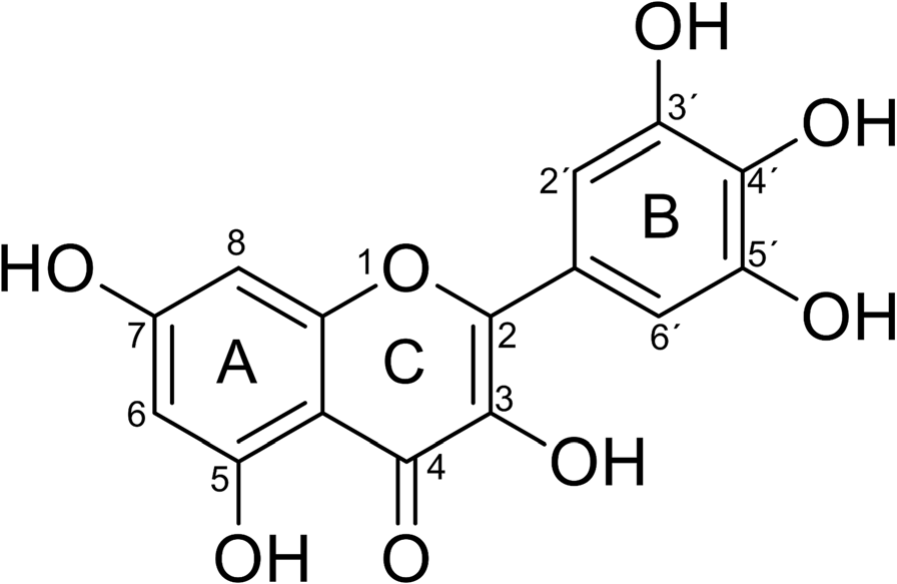
Molecular structure of Myricetin

Polyphenols are a wide group of plant-derived molecules resulting from secondary metabolism, ubiquitously distributed in vegetable kingdom where they display different activities such as protective effect against UV rays, bacteria, virus and fungi infections, modulation of plant hormones, enzyme inhibition and pollinator attraction [1]. In nature, there are a plethora of different polyphenols that can be classified in the following main classes: simple phenolic acids (e.g. gallic, vanillic, syringic, *p*-hydroxybenzoic), hydroxycinnamic acid derivatives (such as caffeic acid, *p*-coumaric, ferulic, sinapic), flavonoids, stilbenes and lignans. The largest common class of polyphenols present in human diet is represented by flavonoids [2, 3]. Chemically flavonoids are classified in flavans, flavones, flavonols, and anthocyanidins [4]. Among the flavonols, myricetin, a 3,3′,4′,5,5′,7-hexahydroxyflavone, possess one of the most hydroxylated structures (Fig. 1). The solubility of myricetin in water is poor (16.6 μg/mL) but increases when deprotonated in basic aqueous media and in some organic solvents (dimethylformamide, dimethylacetamide, tetrahydrofuran and acetone) [5]. The chemical stability of myricetin is pH and temperature dependent [6]. Depending on the environment conditions, myricetin can exert, in vitro, both a potent antioxidant and a pro-oxidant effect. Buchter et al. [7] attributed its direct antioxidant action to several structural elements. On the other hand, Chobot and Hadacek [8] demonstrated the pro-oxidative properties of myricetin to molecular oxygen reduction to reactive oxygen species (ROS) and iron (III) to iron (II) and also highlighted the ability of myricetin to serve as a substitute for ascorbic acid, albeit less efficiently.

Myricetin is mainly present in the glycoside form (*O*-glycosides), in vegetables, fruits, nuts, berries, herbs, plants together with beverages, such as tea, wine, fruit and medicinal plants [9–15]. There are numerous factors that can influence myricetin levels in plant foods such as genetic and environmental factors, germination, and ripeness degree, variety, seasonal variation, and storage, processing and cooking. The estimate of total flavonoid intake is difficult to calculate, as appropriate tables of food composition are not yet available. However, reliable data on daily flavonoid intake in a population are needed to develop proper dietary recommendations and even for correct data interpretation from intervention studies. The Flemish Dietetic Association database determined an average daily intake of myricetin of 2.2 ± 2.5 mg Mullie et al. [16]. In a Korean adult population, Jun et al. [17] estimated an average intake of 0.8 mg/day representing about 1–2% of flavonol subclass, while a mean intake of myricetin 2 mg/day ranged from 1 to 4 mg/day in adults (18 to 64 years) in the European Union was reported by Vogiatzoglou et al. [18]. The knowledge on habitual flavonoids consumption is also crucial to determine their possible impact on human health. Myricetin exhibited antioxidant properties and free radical-scavenging effects [19]. These activities seem to support a wide range of beneficial outcomes including, anti-platelet aggregation, antihypertensive, immunomodulatory, anti-inflammatory, anti-allergic, analgesic, anticancer actions and so on [6, 20–25]. The main goal of the present review is to provide new insights on myricetin preclinical pharmacological activities, and its role in selected clinical trials.

## Myricetin in plants

Myricetin glycosidies include myricetin-3-*O*-(4″-acetyl)-α-l-arabinopyranoside, myricetin-3-*O*-(3″-acetyl)-α-l-arabinopyranoside, myricetin-3-*O*-β-d-galactopyranoside, myricetin-3-*O*-α-l-rhamnopyranoside, myricetin-3-*O*-β-d-xylopyranoside, myricetin-3-*O*-α-l- arabinofuranoside, myricetin-3-*O*-(6″-galloyl)-β-d-galactopyranoside [26], myricetin-3-*O*-(3″-*O*-galloyl)-α-l-rhamnoside, myricetin-3-*O*-(2″-*O*-galloyl)-α-l-rhamnoside, and myricetin-3-*O*-α-l-rhamnoside [27].

The first time myricetin was identified was in plants of the Myricaceae, *Comptonia peregrina (L.) Coult. and later Morella cerifera* (L.) Small [28, 29]. The myricetin concentration in the plants such as *Rosa canina* L. (rosa hip), *Urtica dioica* L. (nettle), and *Portulaca oleracea* L. (purslane) found between 3 and 58 mg/kg [13].

Myricetin was isolated from *Polygonum bellardii* All. (Polygonaceae) as yellow needles (50 mg) from aerial parts using MeOH extract [30]. Previously, a prescreening of leaves of 28 polygonaceous plants was estimated that myricetin glycosides were relatively rare consituents [31]. *Trigonella foenum-graecum* L. gemmo-modified extract had the richest content in myricetin (830 mg/kg), followed by *Euphorbia tirucalli* L. (821 mg/kg), rhizomes of *Cyperus rotundus* L. (702 mg/kg) and seed extract of *T. foenum-graecum* (547 mg/kg). *C. rotundus* gemmo-modified extracts contained 104 mg/kg myricetin [10]. The highest level of myricetin content has been identified in the strawberry and spinach [9]. Species of *Anacardium* and *Mangifera* (Anacardiaceae) found to have high levels of hydroxylated compounds like myricetin, gallic acid, proanthocyanidins and flavonols. In *Marantodes pumilum* (Blume) Kuntze (Primulaceae) were identified quercetin, myricetin, kaempferol, catechin and epigallocatechin [32].

The most common sources of myricetin are vegetables, fruits, nuts, berries and tea [33]. Myricetin-rich foods are listed in Table 1 based on the USDA Food Database (compiled data from all fruits and vegetables that contain information on myricetin concentration) [34]. In black fruits the quantities varied between 14 and 142 mg/kg [12]. Myricetin is the most abundant flavonol of black currant, and its quantity varied significantly among black currant cultivars [35]. At the same time, honey is also a source of flavonoids, especially myricetin. The HPLC analyses of honeys from Australian *Eucalyptus* have shown that the flavonoids myricetin, quercetin, tricetin, kaempferol and luteolin exist in all honeys. Myricetin was found in range from 29.2–289.0 μg/100 g honey [36]. In grapes, flavonol glycosides from the following aglycons have been identified: myricetin (3′,4′,5′-triOH), laricitrin (3′-MeO analog of myricetin) and syringetin (3′,5′-diMeO analog of myricetin), quercetin and kaempferol [37]. The simultaneous presence of these aglycons was detected in different types of red wine *Vitis vinifera* L. grapes [38], while in white wine, only quercetin, kaempferol and isorhamnetin were detected [37].

**Table 1:** Myricetin (mg/100 g) rich foods [34]

Cranberry	6600
Dock	5700
Sweet potato leaves	4400
Chard, swiss	3100
Broadbeans, immature seeds	2600
Rutabagas	2100
Garlic	1600
Blueberry	1300
Peppers, hot chili, green	1200
Blackberry	700
Lotus root	600
Lemon	500

Source: USDA Food Database (compiled data from all fruits and vegetables that contain information on myricetin concentration)

## Preclinical pharmacological activities of Myricetin

Myricetin displays multiple preclinical biological effects [19]. Thus, in the following subsections, the antimicrobial, antioxidant, neuroprotective, antidiabetic, anticancer, immunomodulatory, cardioprotective, analgesic, anti-hypertensive and wound healing potential of myricetin are briefly discussed and summarized.

### Antimicrobial activities

Antimicrobial mechanism of flavonoids may involve membrane disruption, inhibition of cell envelope synthesis, inhibition of nucleic acid synthesis, inhibition of bacterial virulence and quorum sensing, which impairs their ability to form biofilms, inhibition of efflux pumps, and inhibition of NADH-cytochrome C reductase activity and ATP synthase [39, 40]. Myricetin inhibited *Escherichia coli* DNA gyrase (IC_50_ 1.18 mg/dL) [41], and DnaB helicase (IC_50_ 11.3 μM) [42], and cellular DNA and RNA polymerases [43].

Myricetin showed a significant antimicrobial activity against foodborne pathogens in terms of minimum inhibitory concentration (MIC, mg/mL) <15.0, <15.0, <20.0, <10.0 at 24 h and <20.0, <20.0, <15.0, <5.0 at 60 h incubation for *Escherichia coli, Salmonella paratyphi, Salmonella cholerasuis,* and *Salmonella enteritidis*, respectively [44]. The compound myricetin revealed curli-dependent *E. coli* biofilm formation inhibition (IC_50_ = 46.2 μM), curli contributes to the robustness of *E. coli* biofilms [45].

At 100 μM concentration, myricetin exhibited in vitro anti-HIV activity in cell cultures: TZM-bl (> 87%; IC_50_ 20.43 μM), PBMC (86%; IC_50_ 4.49 μM, 3.23 μM), and H9 cell (≥86%; IC_50_ 22.91 μM, 1.76 μM) [46]. Myricetin exhibited the highest anti-HIV reverse transcriptase activity (> 49%, IC_50_ 203.65 μM) at the concentration of 100 μM [46].

Yadav et al. [47] demonstrated the anti-tubercular activity of 15 selected flavonoids including myricetin and their structure–activity relationships were evaluated against *Mycobacterium tuberculosis* H37Rv strain radio-metrically. Myricetin was found to be active against *M. tuberculosis,* with a MIC of 50 μg/mL, and structure–activity relationships authenticated their anti-tubercular potential due to the presence of hydroxy groups in their structure.

The inhibitory activity of the compounds were evaluated against DNA gyrase from *E. coli* by DNA supercoiling. Mean antibacterial activity in terms of MIC and IC_50_ were 142 μg/mL and 1.18 mg/mL respectively. The structure-activity relationship analysis suggests that, the presence of hydroxyl and substitution in the ring A and B position are essential for the best inhibitory effects [41].

The inhibitory effect of myricetin on severe acute respiratory syndrome-coronavirus (SARS-CoV) helicase, nsP13, and hepatitis C virus (HCV) helicase, NS3h was also assessed [48]. Myricetin was found to inhibit SARS-CoV helicase protein by affecting the ATPase activity (IC_50_ 2.71 μM), however, it failed to affect the ATPase activity of the HCV NS3 helicase.

DeSouza and Wahidullah [49] reported the antimicrobial activity on *E. coli, Klebsiella pneumoniae, Proteus mirabilis, Pseudomonas aeruginosa, Salmonella typhi, Shigella flexneri, Staphylococcus aureus, Vibrio cholerae* and myricetin showed the best activity against *P. aeruginosa* (MIC 1.5 μg/mL). Gendaram et al. [50] reported the myricetin antibacterial effect against *S. aureus* by the disc diffusion method (300 μg/disc, inhibition zone 9 mm) but reported no antibacterial activities against *P. aeruginosa, E. coli, Enterococcus faecalis,* or *Micrococcus luteus*. However, at 100 μM concentration, myricetin did not exhibit antimicrobial activity on Gram-positive bacteria but showed inhibitory activity against sortase A (SrtA) from *S. aureus* (92%; IC_50_ 4.63 μM) [51]. In vitro antimicrobial activity of six natural phytochemicals including myricetin (alone and with combination) were evaluated against five strains of *P. aeruginosa* by using a time-kill assay*.* The compound showed the MIC as 500 μg/mL against all five strains of *P. aeruginosa* [52]. Other reports of the compound based on antimicrobial and antiviral studies are presented in Table 2.

**Table 2:** Antimicrobial profiling of the compound myricetin

Strains	Results	References
**Antiviral**		
HIV Reverse Transcriptase	0.08 ^a^	[43]
HIV Reverse Transcriptase, Moloney murine leukemia virus	0.08 ^b^	[53]
**Antimicrobial**		
**Gram positive**		
*Actinomyces viscosus*	20 ^b^	[54]
*Burkholderia cepacia*	>512 ^b^	[55]
*Corynebacterium diphtheriticum*	18.2 ^e^	[56]
*Enterococcus faecalis*	17.0 ^e^	[56]
*Enterococcus faecalis* 2400	17.0 ^e^	[56]
*Enterococcus faecium*	16.8 ^e^	[56]
Methicillin-resistant *Staphylococcus aureus*	256 ^b^	[55]
*Staphylococcus aureus* ATCC6538p	> 300 ^c^	[57]
*Staphylococcus aureus*	> 2000 ^b^	[58]
*Staphylococcus epidermidis* ATCC14490	64 ^b^	[55]
*Staphylococcus epidermidis*	> 2000 ^b^	[58]
*Staphylococcus epidermidis*	17.4 ^e^	[56]
*Staphylococcus saprophyticus*	17.6 ^e^	[56]
*Streptococcus mutans*	20 ^b^	[54]
*Streptococcus pneumoniae* 49	128 ^b^	[55]
*Streptococcus pneumoniae*	17.4 ^e^	[56]
*Streptococcus pyogenes*	16.4 ^e^	[56]
Vancomycin-Resistant Enterococci (VRE)	512	[55]
**Gram negative**		
*Burkholderia cepacia*	64 ^b^	[55]
*Enterobacter aerogenes*	256 ^b^	[55]
*Escherichia coli*	> 2000 ^b^	[58]
*Escherichia coli* WT	12.2 ^e^	[56]
*Escherichia coli* BU40	12.6 ^e^	[56]
*Escherichia coli* FPL5014	11.6 ^e^	[56]
*Escherichia coli* DnaB helicase	11.3 ^d^	[42]
*Klebsiella pneumoniae* ATCC13883	64 ^b^	[55]
*Klebsiella pneumoniae*	128 ^b^	[59]
*Klebsiella pneumoniae*	> 2000 ^b^	[58]
*Klebsiella pneumoniae*	16.6 ^e^	[56]
*Porphyromonas gingivalis*	2500 ^b^	[54]
*Prevotella intermedia*	1250 ^b^	[54]
*Proteus mirabilis*	16.5 ^e^	[56]
*Pseudomonas aeruginosa* ATCC27853	256 ^b^	[55]
*Pseudomonas aeruginosa*	> 2000 ^b^	[58]
*Pseudomonas aeruginosa* PAO286	15.6 ^e^	[56]
*Salmonella paratyphi* A	14.4 ^e^	[56]
*Salmonella paratyphi* B	14.4 ^e^	[56]
*Salmonella typhi*	14.4 ^e^	[56]
*Shigella dysenteriae*	15.5 ^e^	[56]
*Shigella flexneri*	13.4 ^e^	[56]
*Shigella sonnei*	14.6 ^e^	[56]
**Anti-chlamydial**		
*Chlamydia pneumoniae*	29 ^c^	[60]

Microbial strain is inserted when microbial type is repeated and information available

^a^Ki (μM)

^b^minimum inhibitory concentration (MIC, μg/mL)

^c^MIC (μM)

^d^half maximal inhibitory concentration (IC_50_, μM)

^e^zone of inhibition (ZOI, mm) for 100 μL of 0.5 mg/mL myricetin

### Antioxidant activities

Plant-based compounds considered as natural antioxidants have attracted a large number of communities of scientist, researchers, industries and traditional healers for their health-promoting characteristics. The antioxidant potential of myricetin has been reported by several authors in the last few decades.

Hou et al. [61] studied the antioxidant effect of HS15-Myr micelles and independent myricetin by using FRAP (ferric reducing antioxidant power) and ABTS (2,2′-azino-bis(3-ethylbenzothiazoline-6-sulphonic acid) assays. The ABTS assay displayed an improved value from 22.20 to 41.77% in HS15-Myr micelles and 0 to 6.12% in independent myricetin at two different concentrations and incubation periods. The FRAP assay also presented an improved value from 1.27 to 8.94 mM Fe^2+^/g in HS15-Myr micelles and 13.63 to 16.33 mM Fe^2+^/g in independent myricetin at two different concentrations and incubation periods. Myricetin in HS15-Myr micelles exhibited in both assays stronger antioxidant effects when compared to independent myricetin.

Barzegar [62] reported the ROS-protection efficiency of the compound myricetin in a cell-free and cell-based system. A low concentration of compound significantly inhibited intracellular ROS production and also protected cells against toxicity induced by peroxide compounds.

Guitard et al. [63] reported that, myricetin is more efficient than α-tocopherol and synthetic antioxidants on preservation of omega-3 oils. Other studies on antioxidant potential of the compound are presented in Table 3.

**Table 3:** Antioxidant activities of myricetin

Assay	Model	Results	Ref.
Density functional theory	in silico	The bond dissociation enthalpy computed and the compound showed ionization potentials 161.4 kcal/mol.	[64]
Antioxidant response element (ARE) activation	in vitro	Activates Nrf2 antioxidant response element pathways and is involved in myricetin-induced expression profiling in hepatic cells.	[65]
Deoxyribose degradation	in vitro	Significant antioxidant activity (complex with iron) in the presence of ascorbic acid.	[8]
DPPH	in vitro	Myricetin/HP-β-CD inclusion complex formation enhances antioxidant activity of drugs.	[66]
DPPH	in vitro	Significant RSA dose-dependently	[50]
DPPH, ABTS	in vitro	Inhibition activity from 13.3 to 99.8% at doses of 0.03 to 1 mg/ml during 5 to 20 min.	[67]
DPPH, FRAP	in vitro	High RSA in DPPH assay, and intermediate ferric reducing ability in FRAP assay.	[68]
DPPH, FRAP, ABTS	in vitro	Mean activity for FRAP (27.2, 26.7) mmol Fe^2+^/L, DPPH (7.9, 9.3) mmol TEAC/L, and ABTS (9.3, 11.5) mmol TEAC/L.	[69]
DPPH, FRAP, ORAC	in vitro	EC_50_ value of DPPH, FRAP and ORAC assays were recorded as 7.60 μg, 8.86 and 12.99 mmol Trolox equivalents per gram.	[70]
DPPH, TPTZ, superoxide	in vitro	Myricetin and its derivatives showed IC_50_ value from 1.82 to 3.27 μg/mL in DPPH assay and 1.86 to 3.83 μg/mL in superoxide assay however, 1.38 to 2.89 μM equivalent to Fe^2+^ /mL for TPTZ assay.	[71]
H_2_O_2_	in vitro	Increases hydrogen peroxide resistance in *Saccharomyces cerevisiae.*	[72]
DPPH, ROS	in vitro	21–54% scavenging activity in DPPH assay (5–10 μg/mL) and 35–73% intracellular ROS scavenging activity (1–10 μg/mL). Significantly inhibits H_2_O_2_-induced cell death and activated antioxidant enzymes.	[73]
NO	in vitro	Mean scavenging activity compared to hydrophilic antioxidants.	[74]
ROS	in vitro	Inhibits peroxynitrite-mediated DNA damage in primary astrocytes at 5 μM.	[75]
ROS	in vitro	The IC_30_ value for inhibitory effect on triglyceride and ROS were recorded as > 150 μM and 122.7 μM.	[76]
ROS	in vitro	Inhibits H_2_O_2_-induced cell death and increases cell survival (65%).	[77]
DCFH-DA	in vivo	Inhibits ROS production in normal individuals and in patients with sickle cell anemia.	[78]

*ABTS 2,2′* azino-bis(3-ethylbenzothiazoline-6-sulphonic acid, *ARE* antioxidant response element, *DCFH-DA* dichloro-dihydro-fluorescein diacetate, *DPPH* 2,2-diphenyl-1-picrylhydrazyl, *FRAP* ferric reducing antioxidant power, *NO* nitric oxide, *ORAC* oxygen radical absorbance capacity; ROS reactive oxygen species, *RSA* radical scavenging activity, *TEAC* trolox equivalent antioxidant capacity, *TPTZ* tri-pyridyl triazine

### Neurobiological activities

**Fig. 2:**
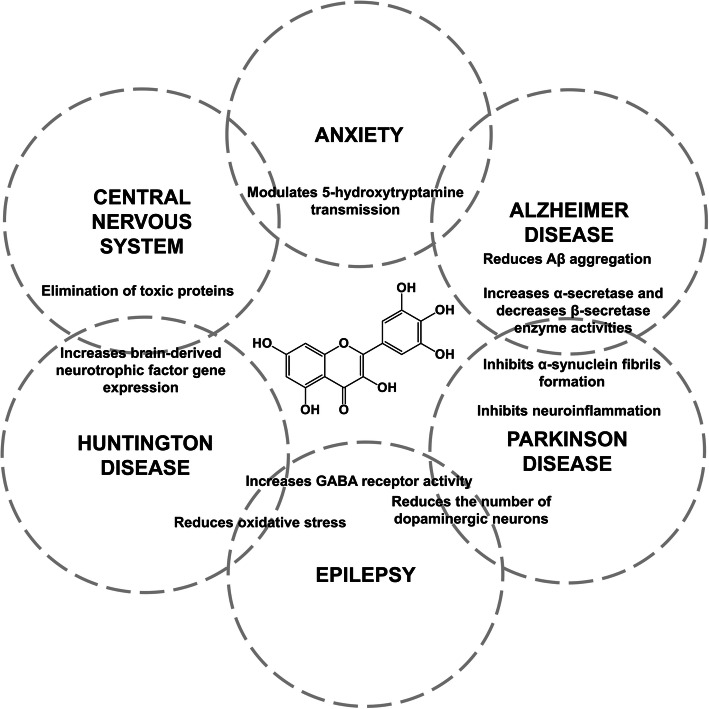
Main mechanisms and activities of myricetin as neuromodulator

Natural flavonoids have exerted positive impacts on body through affecting multiple cell systems and modulating the activity of various pathways to reduce cognitive decline and neuronal dysfunction [79]. Myricetin is one of such flavonoids, and multiple studies have been conducted to assess the neuroprotective effects of this compound and its interaction with brain receptors (Table 4). The main mechanisms are shown in Fig. 2.

**Table 4:** Neurobiological effects produced by myricetin

Model	Results	Ref.
**Anxiety**		
In vitro and in vivo	Dose-dependent reduction in lithium-induced head twitches and anxiolytic activity by altering 5-hydroxytryptamine transmission.	[80]
**Alzheimer disease**		
In vitro	Pro-oxidant agent and reduced the formation of ordered amyloid beta (Aβ)42 aggregation.	[81]
In silico	Destabilizes the β-sheet ordered amyloid oligomers formed by the undecapeptide Aβ (25–35) model.	[82]
In vitro	Marked modulation of metal-induced Aβ aggregation, more than metal-free Aβ aggregation. Increase cell survival rate of Aβ (with metal ions).	[83]
In vitro	Increases α-secretase (ADAM10) enzyme activity and decreases of β-secretase (BACE-1). It also exerts neuroprotective activity against Aβ (1–42) with multifunctional role in counteracting AD progress.	[84]
In vitro	Dose-dependent inhibition of α-synuclein fibrils formation and destabilization (EC_50_ = 0.21–1.8 μM).	[85]
In vitro	Dose-dependent inhibition of Aβ fibrils formation from fresh Aβ (1–40) and Aβ (1–42). The EC_50_ value for formation, extension and destabilization Aβ fibrils ranges from 0.13–1.8 μM.	[86]
In vivo	Increases the number of hippocampal CA3 pyramidal neurons and survival in a rat model (10 mg/kg). Improved learning and memory in a rat model with AD.	[87]
**CNS**		
In vitro	Reduces the aggregation of different abnormal proteins and eliminates various toxic proteins related to neurodegenerative diseases. Improves physiological functions of Hsp70 molecular chaperone and reduces mis-folded proteins.	[88]
In vitro and in vivo	Increases GABA receptor activity via calcium channel/ CaMK-II dependent mechanism, which is distinctively different from that of most existing benzodiazepine binding site agonists of GABA receptor.	[89]
In vivo	Increases mRNA for brain-derived neurotrophic factor (BDNF) in the hippocampus of male C57BL/6 mice at 10 and 20 mg/kg (7 days).	[90]
In vivo	Increases BDNF concentrations in the hippocampus of male C57BL/6 mice at 50 mg/kg (21 days).	[91]
In vivo	Enhances expression and activity of ERK1/2-CREB pathway and Na^+^, K^+^-ATPase while reduces oxidative stress level in hippocampus. Improves learning and memory when compared with D-galactose.	[92]
**Epilepsy**		
In vivo	Reduces seizure severity and mortality rates in mouse models and signaling pathways (BDNF-TrkB) and regulates GAD65/GABA with MMP-9 expression.	[93]
**Huntington disease**		
In vivo	Interacts with RNA, especially CAG motif, and decreases the huntingtin protein translation and sequestration. Reduces cytotoxicity in HD and other polyQ disease models.	[94]
**Parkinson disease**		
In vitro	Suppresses intracellular ROS production, re-establishes mitochondrial trans-membrane potential, and inhibits MKK4 and JNK activation.	[95]
In vitro and in vivo	Inhibits activation of microglia (neuroinflammation), expression of pro-inflammatory mediators and reduces the number of dopaminergic neurons.	[96]
In vivo	Dose-dependent delay in climbing ability loss, but increases the life span of flies expressing human α-synuclein in brain.	[97]
In vivo	Prevents the loss of dopaminergic neurons and dopamine content in brain of Parkinson flies.	[98]
In vivo	Dose-dependent inhibitory activity on α-synuclein aggregation.	[99]
In vivo	Diminishes dopamine neuron degeneration, which is induced by 6-hydroxydopamine and 1-methyl-4-phenyl-pyridinium in substantia nigra-striatum.	[100]

Aβ amyloid beta, CNS central nervous system, BDNF brain-derived neurotrophic factor

### Antidiabetic activities

Myricetin antidiabetic activity has been reported by several authors in the last few years and limited reports are also available on its anti-obesity activity but in this review, we focused on only its antidiabetic potential. Karunakaran et al. [101] reported the in vitro effect of myricetin on high glucose-induced β-cell apoptosis, possibly via cyclin-dependent kinase 5 (CDK5) inhibition. Data revealed that myricetin (20 μM) significantly protect β-cells reducing apoptosis in INS-1 cells and rat islets that were incubated with glucose at the concentration of 30 mM for 24 and 48 h, respectively. Docking studies predicted myricetin inhibited activation of CDK5.

The effect of myricetin was evaluated in diabetes mellitus-associated kidney injuries and dysfunction in an experimental mouse model with diabetes mellitus induced by 5 consecutive injections of low-dose streptozotocin (STZ) [20]. The data revealed that myricetin (orally twice a day, 100 mg/kg/day, for 6 moths) inhibited the IκBα/NF-κB pathway, with this pathway being independent of nuclear factor erythroid 2-related factor (Nrf2) regulation. It was also reported that myricetin activates glucagon-like peptide 1 receptor (GLP-1R) and its long-term oral administration (200 mg/kg, for 40 days) validates its glucoregulatory effects [102].

Insulin’s metabolic action is mediated via the activation of phosphatidylinositol 3-kinase (PI3K) and its downstream effectors, the protein kinase B (PKB/Akt) kinases [103]. In contrast, AMP-activated protein kinase (AMPK) signal pathway is likely to mediate the effect of insulin-independent stimuli for glucose uptake in muscle [104]. In an in vitro study, myricetin enhanced Akt and AMPK protein activity, encouraged glucose uptake and reduced insulin resistance [105]. The mechanisms of myricetin for improving insulin-sensitive tissue might be the amelioration of impaired signaling intermediates downstream of insulin receptors through enhancing the secretion of β-endorphin, which in turn led to the activation of peripheral μ-opioid receptors [106, 107]. Then, myricetin affects insulin receptor phosphorylation, insulin receptor substrate-1 (IRS-1), the p85 regulatory subunit of PI3K, Akt and Akt substrate of 160 kD, with subsequent effects on glucose transporter 4 (GLUT4) translocation [108].

Other previous studies on antidiabetic potential of the compound are shown in Table 5.

**Table 5:** Previous studies on preclinical antidiabetic potential of myricetin

Compound / Plant species	Model	Results	Ref.
Myricetin	in vivo	Enhanced enzymatic and non-enzymatic antioxidant defense system and showed protective effects against oxidative damage in liver and kidney of streptozotocin-cadmium-induced diabetic model.	[109]
Myricetin	in vivo	Inhibitory activity against α-glucosidase (IC_50_ = 414 μM) in dose dependent manner.	[110]
Myricetin	in vivo	Anti-hyperglycemic and renoprotective effects at 1.0 mg/kg.	[111]
Myricetin	in vivo	Improved and re-established renal functions and activities of the glutathione peroxidase and xanthine oxidase enzymes in diabetic rat model.	[112]
Myricetin	in vivo	Antidiabetic activity against t-BHP-induced oxidative stress.	[113]
Myricetin	in vivo	Reduced glycemia in diabetic rats up to 50% after 2 days of treatment at 3 mg/12 h.	[114]
Myricetin	in vivo	Stimulated lipogenesis in rat adipocytes and enhanced the stimulatory effect of insulin (EC_50_ = 65 μM).	[115]
Myricetin	in vitro	Inhibited intestinal α-glucosidase (29%) and porcine α-amylase (64%) with IC_50_ vale of 0.38 mM.	[116]
*Abelmoschus moschatus* Medik. (aerial part)	in vivo	Improved insulin sensitivity in rats.	[117]
*Ampelopsis grossedentata* (Hand.-Mazz.) W.T. Wang (leaves)	in vivo	Inhibitory activity against α-glucosidase (IC_50_ = 319.3 μM).	[118]
*Azadirachta indica* A.Juss. (leaves)	in vivo	Enhanced insulin signaling pathway and glucose utilization in skeletal muscle.	[119]
*Hovenia dulcis* Thunb. (seeds)	in vitro	Inhibited intestinal α-glucosidase with IC_50_ = 3 μg/mL and α-amylase with IC_50_ = 662 μg/mL.	[120]
*Myrtus communis* L. (leaves)	in vivo	Significant antidiabetic activity in diabetic models.	[121]
*Syzygium cumini* (L.) Skeels (seeds)	in vitro	Inhibitory activity against α-glucosidase (IC_50_ = 1.7 μg/mL) and α-amylase (IC_50_ = 7.62 μg/mL).	[122]
*Syzygium malaccense* (L.) Merr. & L.M.Perry (leaves)	in vitro	Inhibitory activity against α-glucosidase (IC_50_ = 15.52 μg/mL) and α-amylase (IC_50_ = 147.30 μg/mL).	[123]

### Anticancer activities

Cancer is responsible for second highest cause of death across the globe [124, 125]. It has been reported that number of death due to this devastating disease would expand to over 13 million by 2030 [126, 127]. Laboratory and clinical studies have reported that myricetin from natural sources exerts promising effects against various types of cancer [19, 21]. The dietary compound myricetin also has the potential to inhibit key enzymes involved in cancer initiation and growth.

Myricetin has presented cytotoxic activity in human colon cancer cells. Kim et al. [21] demonstrated that myricetin significantly induces the Bcl2-associated X protein (BAX)/Bcl2 ratio, and induces apoptosis of HCT-15, in a dose-dependent manner (5 to 100 μM). This study suggested that myricetin can be utilized for the design of therapeutic agents against human colon cancer. Myricetin also acts as a potent inhibitor of human flap endonuclease 1 (hFEN1) protein (IC_50_ 690 nM), based on inhibitory mechanisms, molecular docking, and cancer cell-based assays [128]. The hFEN1 protein is a functional member of the 5′-nuclease superfamily. By chemical nature, hFEN1 is a metal ion-dependent and structure-specific nuclease and also instrumental in DNA replication and repairing processes. Molecular docking studies revealed that ring A of myricetin compound, including 4-keto and 5-OH, was found stretched towards the two divalent metal ions. Both metal ions are critical as they seem to interact with Arg100 and Lys93 amino acids through hydrogen bonds. These interacted residues are well known for their critical interplay in hFEN1’s activity during human colon cancer.

Myricetin has also been shown to protect against ovarian cancer through suppressing ovarian cancer cell angiogenesis [129]. Anti-angiogenic effects of myricetin (5 to 20 μM) assessed through in vitro (HUVEC) and in vivo (CAM) models revealed that this compound significantly inhibits angiogenesis induced by OVCAR-3 cells. In SKOV3 human ovarian cancer cells, myricetin inhibited viability and induced apoptosis (40 μg/mL, time-dependent manner) through endoplasmic reticulum stress and DNA double-strand breaks [130]. Zheng et al. [131] stated that in A2780 and OVCAR3 ovarian cancer cells, the dietary flavonoid myricetin induced significant cytotoxicity (IC_50_ = 25 μM). In a recent study, Tavsan and Kayali [132] reported that myricetin suppressed ovarian cancer cell growth, induced apoptosis, arrested cell cycle and also had the potential to inhibit cell invasion in a significant manner (IC_50_ = 184 μM A2780, 32 μM OVCAR-3, 3.3 μM SKOV3, and > 500 μM OSF). Thus, it can be concluded that myricetin has enough potential to cope with ovarian cancer in a significant manner.

Myricetin has potent anticancer-promoting activity against skin cancer. It was found capable of inhibiting neoplastic cell transformation and mitogen-activated protein kinase 1 (MEK1) activity (myricetin 1 or 5 μM) [133]. Molecular interaction between myricetin and MEK1 suppressed MEK1 activity leading to downstream signaling to the ERK/p90RSK/AP-1 pathway. In another study, myricetin has been presented as a potent chemo-protective agent against skin cancer [134]. Myricetin can bind directly to central kinases including PI3-K, Akt, JAK1, Raf1, MEK1, MKK4, and Fyn, which regulate multiple cell signaling pathways in cancer cells. Myricetin inhibited 12-*O*-tetradecanoylphorbol-13-acetate (TPA)- and epidermal growth factor (EGF)-induced cell transformation by 76 and 72%, respectively at 10 μM concentration. Sun et al. [135] recently reported that myricetin has anticancer activity against skin cancer A431 cell lines, by inducing apoptosis and cell cycle arrest and exhibited low toxicity.

An earlier in vitro study demonstrated the anti-metastatic effect of myricetin in human lung adenocarcinoma A549 cells [136]. This study revealed that myricetin (5 to 20 μM) suppresses adenocarcinoma A549 cell invasion and migration through inhibition of the ERK pathway in a time-dependent manner. Along with a combination of radiotherapy, myricetin was found responsible to enhance the tumor radio-sensitivity of lung cancer A549 and H1299 cells through significant suppression of cell-surviving fraction and proliferation [137]. Wang et al. [138] found that the combination of myricetin with 5-fluorouracil chemotherapy has the potential to enhance tumor chemo-sensitivity of esophageal cancer EC9706 cells. Sun et al. [139] investigated the function of myricetin phytochemical against human T24 bladder cancer in a dose- and time-dependent fashion, and stated that myricetin significantly inhibits both T24 cancer cells viability and proliferation (IC_50_ = 85 μM).

### Immunomodulatory activities

The preclinical immunomodulatory effects of myricetin have also been increasingly reported. Ghassemi-Rad et al. [140] concluded that myricetin has the potential to inhibit T-lymphocyte activation in a mouse model through bead-immobilized anti-CD3 and anti-CD28 monoclonal antibodies. This study clarified the mechanism of action and reported the suppressive effect of myricetin on T lymphocytes mediated through extracellular H_2_O_2_ generation. In mouse primary macrophages and RAW264.7 monocytic cell-line, this phenolic compound was found to inhibit the lipopolysaccharide (LPS)-induced interleukin (IL)-12 production in a significant manner through down-regulation of NF-κB binding activity [22]. In isolated rat aortic rings, myricetin induced endothelium-dependent contractile responses at 50 μM. Earlier, Jiménez et al. [141] reported that, in cultured bovine endothelial cells, this compound is responsible for stimulating the production of cytosolic free calcium. In a dose-dependent manner, myricetin inhibited the secretion of a potent T cell growth factor, namely IL-2 protein from mouse EL-4 T cells, activated with phorbol 12-myristate 13-acetate (PMA) plus ionomycin [142]. In vitro evidence demonstrated that at 5–100 μM, myricetin inhibits CD69 expression and lymphocytes proliferation in a mouse model. Moreover, an in vitro investigation revealed that myricetin significantly effects IL-2 expression. However, further in vitro and in vivo investigations are required to explore myricetin as an immunomodulatory agent.

### Cardioprotective activity

Previous studies have demonstrated that myricetin also has beneficial effects on the human vascular system [23]. In human umbilical vein endothelial cells, myricetin (100 μM), revealed vasculoprotective effects through changes at the transcriptional level [143]. Myricetin has been presented as a functional agent towards preventing atherosclerosis through inhibition of CD36 cell surface protein and mRNA expression in a significant manner [144]. In isolated and Langendorff-perfused rat hearts, without affecting contractility and relaxation, myricetin elicited coronary dilation [145]. In Triton-treated hyperlipidemic rats, evidence from an in vivo investigation demonstrated that myricetin exerts lipid-lowering activity and suggests that myricetin can be utilized in the treatment of hyperlipidemia and cardiovascular diseases (CVD) [146].

In Wistar rats, myricetin significantly inhibited the effects of histopathological changes of isoproterenol on heart rate, the levels of different cardiac marker enzymes, including lactate dehydrogenase (LDH), creatine kinase (CK), aspartate aminotransferase (AST), superoxide dismutase (SOD) and catalase (CAT), as well changes in vascular reactivity and electrocardiographic patterns [147].

A mechanism-based study by Scarabelli et al. [148] demonstrated that myricetin exerts strong inhibitory activity against signal transducer and activator of transcription 1 (STAT1) activation, and also protects the heart from ischemia/reperfusion-injury. The available genomic and genetics data from preclinical experiments have shown that myricetin is likely to confer the first line of defense against cardiovascular and other associated diseases.

### Analgesic activities

In acetic acid-induced writhing response, formalin-induced paw licking, sedative activity and hot plate test models, myricetin revealed potent analgesic effects, closely related with peripheral analgesia, but not with the opioid system [24]. The compound also produced a significant analgesic effects in a rat model of neuropathic pain, by decreasing spinal nerve ligation-induced mechanical allodynia and thermal hyperalgesia lasting for several hours (0.1–10 mg/kg *i.p.)* [149].

### Antihypertensive activities

The antihypertensive effects of myricetin were evaluated in the deoxycorticosterone acetate (DOCA)-salt-hypertensive rat model. Myricetin reduced systolic blood pressure, vascular reactivity changes and reversed the increased heart rate induced by DOCA. At oral doses of 100 and 300 mg myricetin/kg b.w., the compound displayed antihypertensive propertie in the DOCA rat model of hypertension [25]. In another study, the compound lowered the high blood pressure that was induced by fructose doses of 100 and 300 mg/kg p.o. in rats and reversed sugar-triggered metabolic changes [150].

### Wound healing

The wound-healing effects of myricetin-3-*O*-β-rhamnoside were investigated on three different types of cells, keratinocytes, fibroblasts, and endothelial cells. The compound exhibited significant wound healing activity at 10 μg/mL [151].

## Myricetin in clinical trials

Although the number of clinical studies reporting myricetin health benefits in ailments and disorders is low, the increasing data from preclinical studies have supported its beneficial effects [152, 153].

In a 4-week randomized placebo-controlled clinical trial the effect of 300 mg Blueberin (250 mg Blueberry leaves, *Vaccinium arctostaphylos* L., and 50 mg myricetin, three times per day) on fasting plasma glucose and some other biochemical parameters has been investigated in 42 female volunteers (46 ± 15 years; body mass index, BMI, 25 ± 3 kg/m^2^) with diabetes type 2. The Blueberin treatment significantly reduced fasting plasma glucose from 143 ± 5.2 mg/L to 104 ± 5.7 mg/L. In addition to antidiabetic effects, results showed that Blueberin also possessed pharmacologically relevant anti-inflammatory properties, reduced plasma enzyme levels of alanine aminotransferases (ALT), AST, glutamyltransferase (GGT), and reduced serum C-reactive proteins (CRP) [154]. Emulin™ (250 mg of patented blend of chlorogenic acid, myricetin, and quercetin), when regularly consumed, was able not only to lower the acute glycemic impact of foods, but also to chronically decrease blood glucose levels in type 2 diabetic humans (reductions between 1 and 5%) [155]. This study was performed in 40 male and female with fasting glucose range between 126 to 249 mg/mL and a BMI ≥ 30 kg/m^2^.

Data from different studies also indicate the importance of myricetin as a chemopreventive agent, acting on cell proliferation, signaling mechanisms, apoptosis, angiogenesis, and tumor metastasis [156]. Through the analysis of habitual food consumption of 10,054 participants of Finnish Mobile Clinic Health Examination Survey developed during 1966–1972, Knekt et al. [157] estimated that higher myricetin intakes in men led to lower prostate cancer risk. In a prospective study, Gates et al. [158] analyzed the association between the 5 common dietary flavonoids (myricetin, kaempferol, quercetin, luteolin and apigenin) intake and epithelial ovarian cancer incidence in 66,940 women. No clear association was found between total intake of examined flavonoids and incidence of ovarian cancer (Relative Risk [RR] = 0.75 for the highest versus lowest quintile, 95% confidence interval [CI] = 0.51–1.09; p-trend = 0.02), nor for myricetin intake (RR = 0.72, 95% CI = 0.50–1.04; p-trend = 0.01). However, there was a significant 40 and 34% decrease in ovarian cancer incidence for the highest versus lowest quintile for kaempferol and luteolin intake, respectively [158]. The association between flavonoids and flavonoid-rich foods intake and exocrine pancreatic cancer development within the α-tocopherol, β-carotene cancer prevention study cohort were also examined [159]. Of the 27,111 male smokers with 306 pancreatic cancers, the data obtained suggests that a flavonoid-rich diet may decrease pancreatic cancer risk in male smokers not consuming supplemental α-tocopherol and/or β-carotene. Tang et al. [160] showed that high/increased flavonoids (e.g., myricetin) intake is associated with lower lung cancer risk in their studied population (meta-analysis of 8 prospective studies and 4 case-control studies involving 5073 lung cancer cases and 237,981 non-cases).

The intake of 36 g lyophilized grape powder (rich in flavans, anthocyanins, quercetin, myricetin, kaempferol, and resveratrol) also had a great impact in key risk factors for coronary heart disease (lowered levels of triglyceride, low-density lipoproteins, apolipoproteins B and E) in both pre- and post-menopausal women [161]. The study was performed on 24 pre- and 20 post-menopausal women for 4 weeks. However, wide ranges of clinical studies are still needed on the potential activities of myricetin which have been already indicated through in vitro and in vivo experiments.

## Conclusions

Myricetin is a flavonoid present in many foods that has shown biological activities in numerous studies and has a potential use as a nutraceutical. Its antimicrobial and antioxidant role is widely studied, and numerous studies have shown neurobiological activities and a potential beneficial impact on AD, PD, HD and ALS. Also, preclinical studies have revealed antidiabetic, anticancer, immunomodulatory, anti-cardiovascular, analgesic and antihypertensive activities. These studies investigated the effect of myricetin, pure compound or plant extract rich in this compound. In plant studies, the extracts rich in myricetin always have other flavonoids that have also shown antioxidant activity alone. Nevertheless, new well-designed studies have to be performed to study all of the biological effects described before, as well as pre-clinical studies comparing the effect of myricetin compared to other flavonoids and phytochemicals. In the case of neurological diseases, more in-depth studies have to be designed to show the pre-clinical results.

## Data Availability

Not applicable.

## Abbreviations

ABTS: 2,2′-azino-bis(3-ethylbenzothiazoline-6-sulphonic acid
ALT: Alanine aminotransferases (ALT)
AMPK: AMP activated protein kinase
AST: Aspartate aminotransferase
BAX: Bcl2-associated X protein
CAT: Catalase
CDK5: Cyclin-dependent kinase 5
CI: Confidence interval
CK: Creatine kinase
CRP: C-reactive proteins
CVD: Cardiovascular disease
DOCA: Deoxycorticosterone acetate
EGF: Epidermal growth factor
FRAP: Ferric reducing antioxidant power
GGT: Glutamyltransferase
GLUT4: Glucose transporter 4
GLP-1R: Glucagon-like peptide 1 receptor
HCV: Hepatitis C virus
hFEN1: Human flap endonuclease 1
IL: Interleukin
IRS-1: Insulin receptor substrate-1
LDH: Lactate dehydrogenase
LPS: Lipopolysaccharide
MEK1: Mitogen-activated protein kinase 1
MIC: Minimum inhibitory concentration
Nrf2: Nuclear factor erythroid 2-related factor
PI3K: Phosphatidylinositol 3-kinase
PKB: Protein kinase B
PMA: Phorbol 12-myristate 13-acetate
ROS: Reactive oxygen species
SARS-CoV: Severe acute respiratory syndrome-coronavirus
SOD: Superoxide dismutase
SrtA: Sortase A
STAT1: Signal transducer and activator of transcription 1
STZ: Streptozotocin
TPA: 12-O-tetradecanoylphorbol-13-acetate
RR: Relative risk
